# Supplementary material for: Patterns of Intron Gain and Loss in Fungi
Source: PLoS Biol. 2004 Nov 30;2(12):e422. doi: 10.1371/journal.pbio.0020422 (PMC532390; doi:10.1371/journal.pbio.0020422)
Supplement: Table S1 — Also available at http://genes.mit.edu/NielsenEtAl/. (4.3 MB ZIP). [file pbio.0020422.st001.zip › NielsenEtAl/html/488.html]

AN8172.1.NCU03756.1.MG08189.1.FG07188.1


```
 CLUSTAL W (1.82) Multiple Sequence Alignments - Introns Inserted


Sequence 1: NCU03756.1	673 aa
Sequence 2: MG08189.1	636 aa
Sequence 3: AN8172.1	590 aa
Sequence 4: FG07188.1	733 aa
Alignment Length: 787 aa
Number Identitical Residues: 187 aa
Alignment Score (without introns) 11163


MG08189.1 	MQLPTRPLSRRQDNGEDD---------------SRPLPRDLSHYYSQTTKNRKPSQVKEF
NCU03756.1	MSESSVAYHDGPPRGAD----------------GKPLPLDFTHHLSEVTKRRQASQMKKY
FG07188.1 	MAPSTSSKARSESNATVISSVTATTATKGAALKSLPSPKDLSHLFSIITRNRDQSEIKAF
AN8172.1  	MASLSP-------------------------------PLDLSHHFSLTTKRRAPSAVKDF
          	*   :                                * *::*  *  *:.*  * :* :

MG08189.1 	YKFFQIPGIVNFAGG1LPNLRFFPFDTLEAQTAKPERWTPSESTATNTDSRSNG------
NCU03756.1	YRFFQIPGIKNLAGG1LPNTKFFPFETLEAQTAKPERWQPTPNHPGTDKNKKD-------
FG07188.1 	YKFMQIPGISNFAGG1MPNAQYFPFETLEAQISKPDRWQPTANYPEQVEEPAPEPAPPAP
AN8172.1  	YKYFMIPGIANLAGG1LPNASYFPYDTLEASAAHPQRFP---TIPGGKKAKGP-------
          	*::: **** *:*** :**  :**::****. ::*:*:    . .   .           

MG08189.1 	-------------------------DSRDRSESTHITVPLVAKETDPLRKIDLATALQYG
NCU03756.1	----------------------------PAAEPAHITVPTVSGEANPVKKVDVATALQYG
FG07188.1 	TSGLHGFISHINKKGKNIIHKDNNKEADSHKKPTRIIIPKFVNETDPAKRVDLTTALQYG
AN8172.1  	------------------------------QTSERIVVPKESRSAT-SKKIDITTALQYG
          	                                . :* :*    .:   :::*::******

MG08189.1 	MADGYPPLLSFVRQFTREHLHPDVPYRSGPEVVLTVGSTDGFAKTLELFVDPWSEGKGDD
NCU03756.1	QANGYPPLLDWVRKFTRDHLHPDAPYSGGPDVILTCGSTDGFAKTLEMFVNPWIDG-LND
FG07188.1 	QVTGYPPLLSFIKQFTNKVLYPDIPYKGGVDVILNNGSTDGFSKVLQLLVDPWYEG-LHP
AN8172.1  	TSEGLPPLATFVNQFVREHLHPNIPYAGGPGTLLTTGATDGFSKAIEAFTETWNPD-RDG
          	   * ***  ::.:*... *:*: ** .*  .:*. *:****:*.:: :.:.*  .  . 

MG08189.1 	VRDRPGLLCERFAYGNVLTQARPKGVQVVTVEADSGGMLADGPGGLEDVLANWDDSKGRR
NCU03756.1	PRERPGLLCETFVYGNILSQAEPKGVQIVPIKADENGMLATGEGGLEDVLANWDVSKGKR
FG07188.1 	TSERQGMLCETFVFGNIITQVKPLGINIVPIEIDDQGMVVEGPGGLREVLENWDPENGLL
AN8172.1  	IHLRQGILCEEFVYMNAIQTVKPRGLNIVPVAIDDEGMLPYGPGGLAEVLGNWNHRKGCR
          	   * *:*** *.: * :  ..* *:::*.:  *. **:  * *** :** **:  :*  

MG08189.1 	PHLLYTVT~MGHNPTGIVLDVERRKALYAICSKYDVLIVEDDPYW2YMQFPSA-----AR
NCU03756.1	PHLMYTVT2LGHNPTGFVIPVSRRKEIYAICSRYDVIIVEDEPYW~YLQFPSATAANTAK
FG07188.1 	PHFLYTVT2MGDNPSSGVVGIRRRREIYALASKFDIIIVEDDPYW~FLQFPSA-----AL
AN8172.1  	PHLMYTIT2IGQNPTGGTLSVERRKQIYAICQKYDVIIVEDDPYW~NLQYPSAQ---ELQ
          	**::**:* :*.**:. .: : **: :**:..::*::****:***  :*:***       

MG08189.1 	EEAKSRNLPPPQ-----VTSTYSRSTGYPFLDSLVPSFLSFDVDGRVVRLDTFSKTVAPG
NCU03756.1	ATAKASGLKPSQPEQGGEISKRTVSTGYPFLDSLTPSYISIDVDGRVIRLDTFSKTIAPG
FG07188.1 	HEAKARGYGKPQDT---QAPKPAIKSGYEFIDSLARSYLSMDIDGRVIRLDTFSKTIAPG
AN8172.1  	ARHRNSSVNPALSER--NYNAGRKSSGYKFLDSLVPSYLSIDMDGRVVRLDTFSKTIAPG
          	   :  .   .             .:** *:***. *::*:*:****:********:***

MG08189.1 	CRLGWLTAQPAIIERFVR2ITETSTQQPSGFVQSMIGELVMSSQP---------------
NCU03756.1	CRLGWITAQPELIDRFNR~IAETSTQQPSGFVQSLVAELVMGPQPEAPSSSSSSSSSSST
FG07188.1 	ARLGYITAQPAIIERLTR2IAETGAGQPSGFVQALVAQTLLGPQSS--------------
AN8172.1  	CRLGWITAQPAFIERLAR~IAESSTQAPSGFVQAMVAELILGQQVD--------------
          	.***::**** :*:*: * *:*:.:  ******:::.: ::. * .              

MG08189.1 	TETKTTFLGLRTHKDRTAFT----------GWQMDGWVRWLEGLRGVYEDRMNRVCRILD
NCU03756.1	TATTSSSSGWSLFRSSSSKPQPAKQTPSGEGWQMTGWVRWLEGLRGMYERRMNRMCTILD
FG07188.1 	TLQKFFYLPQSKKPTFT-------------GWKLDGWVRWLEGLRGEYERRMIRMCKTLE
AN8172.1  	DGSADSAKSKDTER----------------AWKMDGWVRWLEGLRAGYEQRMQQMCSGLE
          	                              .*:: **********. ** ** ::*  *:

MG08189.1 	DGKYQLKQG-TPVVPGSHP----LDADWGVITKKKLFEFDWPRGGMFVWLRVFFESHPLW
NCU03756.1	SGSSLVSSS-TRLRATSGSSSVGTPGDWEVITTTPLYTYSWPEGGMFIWLRMHFDTHPLW
FG07188.1 	ENAFQLNSS-----TPSSP-----DSDWNIITKTRLMDFDWPRGGMFLWVRVHFEKHPLY
AN8172.1  	EGKYIALHTRSDEFSLSGN----EEDSWEVLEKVQMYDFAWPTGGMFVWVKVNYESHPLR
          	..        :   . *         .* :: .  :  : ** ****:*::: ::.*** 

MG08189.1 	KAPR--PGK---DGEPAGVMDGGVLSGALLAMLTKKPWLVLVAPGFMFSANEEIQRKDGW
NCU03756.1	QHPLFFPSNGNGEGEKTKVIDGPTISLALMLFLTLKPHLVLVSAGNMFSATSQIREEVGW
FG07188.1 	QAKVG--------RKSLPTIDGPALSKAFLIHSTRAPHLVLGSPGTMFSATPQIRDERGW
AN8172.1  	-----------------QIYGPERLSKALWVHFTQKPQLCLLGPGTIFSPTDEVQAR-GH
          	                    .   :* *:    *  * * * ..* :**.. ::: . * 

MG08189.1 	A~YF-------------------------RLCFAAEAEEAVDPCTQRFV~DGIQRFWKIK
NCU03756.1	Q~YF-------------------------RLCFAAVSEEDVDSGAQRFV~DGVRAFWKIT
FG07188.1 	R2RTTDTAYMSKLPVSIYLAQICDSLMDTRECTDSFSHKEVPSLSLYLG1YRSRIIEYRH
AN8172.1  	Q~YF-------------------------RLCFAAMPADDVVPITQRVV~DGFRSFWQRK
          	                             * *  : . . * . :  .     : :    

MG08189.1 	TVREIEDLLKGFPISSGE-----------------------ASSMNVADQDVGNMGMYMG
NCU03756.1	DARVIEKLVEQK-VDTEE-----------------------VVARVMGQEGVNNLGGWLG
FG07188.1 	SLILKEGIDKSRPQSQPKQLCLQPKFQSRPQQGIPPAQLPFVSAALIVVVGLGYVDRQLN
AN8172.1  	NLDGLE-----GDDGIAQ-----------------------TMEQLQMEPSANFLG--MG
          	     *        .  :                       .        . . :.  :.

MG08189.1 	C------------
NCU03756.1	C------------
FG07188.1 	GLKWVILLFSYLV
AN8172.1  	C------------
          	
```
